# FOMO in the time of coronavirus disease

**DOI:** 10.1017/cem.2020.410

**Published:** 2020-05-28

**Authors:** Dana E. Phillips

**Affiliations:** * Division of Emergency Medicine, Sunnybrook Health Sciences Centre, University of Toronto, Toronto, ON

**Keywords:** COVID-19, emotion, maternity leave, reflection

I have FOMO: *Fear of Missing Out*. Usually, FOMO is a lighthearted reference. It's what you feel on that perfect summer evening when your friends are at a BBQ and you have to work. However, as an emergency medicine physician in the time of coronavirus disease (COVID-19), my FOMO is more complicated. I am on maternity leave and am missing working on the front lines.

Half of my brain feels this is the perfect time *not* to be in the emergency department (ED). A deadly virus is sweeping through the population, and I am safe at home playing peek-a-boo with a cuddly baby. The other half of my brain is wrecked by that FOMO. COVID-19 is hardly a BBQ. Hundreds of thousands of people have died, and front-line workers have taken their lives over the stress of battling this disease. However, the COVID-19 pandemic will likely be the most significant world event of my lifetime, a fabled era my daughter will one day question me about. “Mommy, will you tell me about helping people in the emergency department when there was that bad disease?” I will answer that I missed the pandemic. People were dying, and I was not there to help.

I chose a career in medicine, as cliché as it sounds, because I wanted to “help people.” I have a Rolodex of cases that have validated and reinforced that desire: resuscitating a patient in cardiac arrest and watching him walk out of the hospital five days later; when unable to save a patient, palliating her through her last hour. Channeling those memories used to be empowering, but it now leaves me feeling powerless and suffering pangs of guilt. The skills I worked so hard to cultivate – running a code, providing end-of-life care – go unused as I am sheltered at home with my family.

I've often lain awake, wrestling with the thought of ending my maternity leave early and returning to the front lines. I've stared at the ceiling, tormented by the tug of war between the yearning to spend every minute with my precious daughter and a duty to serve my community. Fortunately, that decision has been made easier by the local numbers. The surge has not happened here, and ED volumes are low. I am not needed.

Daily, I read my hospital's COVID-19 email updates from the safety of my living room. I follow the posts offering encouragements in our physician WhatsApp group. I devour podcasts on how to manage the ventilator for COVID-19 patients. But I have not managed the ventilator of a COVID-19 patient. I have not done a protected intubation. I have not donned personal protective equipment (PPE).

Watching YouTube videos of these new procedures between feedings and diaper changes is not the most effective way for a doctor to keep up to date in a pandemic. Learning is hands-on, and practice makes perfect, so I am becoming a diaper-changing expert as my intubation skills wane. And now physicians are intubating in aerosol boxes and jerry-rigging ventilators to the same circuit! Incredible experiential learning is happening in the ED and, for months, I have been missing from class.

My homeschooled pandemic medical education is also lonely. As major league innovation occurs on the field, I gaze on from the bench. Together, my team matches this formidable opponent and adapts in lockstep to its curve balls. Our once silent departmental text thread is teeming with messages. Posts of colleagues huddled together, barely recognizable in PPE, fill my social media feeds like never before. They are #healthcareheroes. Not just 2 meters apart, I feel a world away, missing the bonds formed through the shared mission of serving the public.

To some, this probably sounds twisted. Why would anyone in her or his right mind want to be on the front lines of a battleground facing a deadly opponent? Shouldn't I feel only relief over the serendipitous timing of my maternity leave? My emotion is not necessarily a *desire* to be in the ED but that I am missing out on something not being there: the camaraderie, the urgency, the gratification of offering my small part in a global crisis.

It is obviously tongue-in-cheek when I call my emotion FOMO. COVID-19 is not a concert or friend's birthday party. I anxiously pour over my calendar, counting the days until my return to the ED, afraid of being infected and, worst of all, infecting my baby. The maternal instinct is to protect your child at all costs, but am I letting down society by protecting her?

When I return to work, I will walk into an ED where medicine is completely different than how I left it. The top of every differential diagnosis will be a disease I have never seen. Something as familiar as the uniform scrubs will be transformed to a complex layering of PPE that I don't know how to put on. I am left behind. I missed out on the simulation training, the tutorials on how to don and doff. I will not be learning *with* my colleagues. I will be learning *from* my colleagues. Fortunately, they are amazing teachers, and I will humbly be their student.

We are all figuring out how to process our emotions in the age of COVID-19. I did not expect to feel anything but grateful for missing out on the pandemic, yet duty – and dare I say curiosity? – has led to my FOMO. Hesitantly, I share these sentiments with those risking themselves on the front lines, embarrassed over feeling sorry for myself not being there.

To those who are not missing out on COVID-19 but facing it head on, working so I can shelter at home with my baby, thank you for your brave, selfless service. Perhaps you would give anything to trade places with me, to escape the front lines for a world where the biggest threat is a fussy, teething infant. But have you ever considered how you would feel *not* being there? And if you couldn't be there, would you have FOMO too?

